# Response to Visceral Leishmaniasis Cases through Active Case Detection and Vector Control in Low-Endemic Hilly Districts of Nepal

**DOI:** 10.4269/ajtmh.21-0766

**Published:** 2022-07-05

**Authors:** Megha Raj Banjara, Anand Ballabh Joshi, Vivek Kumar Singh, Murari Lal Das, Chitra Kumar Gurung, Piero Olliaro, Christine Halleux, Greg Matlashewski, Axel Kroeger

**Affiliations:** ^1^ Central Department of Microbiology, Tribhuvan University, Kirtipur, Kathmandu, Nepal;; ^2^ Public Health and Infectious Disease Research Center, New Baneshwor, Kathmandu, Nepal;; ^3^ Centre for Tropical Medicine and Global Health, Nuffield Department of Medicine, Oxford University, Oxford, United Kingdom;; ^4^ UNICEF/UNDP/World Bank/WHO Special Programme for Research and Training in Tropical Diseases (TDR), World Health Organization, Geneva, Switzerland;; ^5^ Department of Microbiology and Immunology, McGill University, Canada;; ^6^ Freiburg University, Centre for Medicine and Society, Freiburg, Germany

## Abstract

The visceral leishmaniasis (VL) elimination program in Nepal has largely completed the attack phase and is moving toward consolidation and maintenance phases. New VL foci are, however, appearing in Nepal, and therefore new innovative community-centered strategies need to be developed and tested. We conducted early case detection by an index case–based approach and assessed the feasibility, efficacy, and cost of an intervention for sandfly control through indoor residual spraying (IRS) or insecticidal wall painting (IWP) in new and low-endemic districts Palpa and Surkhet. IRS was performed in 236 households and IWP in 178 households. We screened 1,239 and 596 persons in Palpa and Surkhet, respectively, resulting in the detection of one VL case in Palpa. Both IWP and IRS were well accepted, and the percentage reductions in sandfly density after 1, 9, and 12 months of intervention were 90%, 81%, and 75%, respectively, for IWP and 81%, 59%, and 63% respectively for IRS. The cost per household protected per year was USD 10.3 for IRS and 32.8 for IWP, although over a 2-year period, IWP was more cost-effective than IRS. Active case detection combined with sandfly control through IWP or IRS can support to VL elimination in the consolidation and maintenance phase.

## INTRODUCTION

Visceral leishmaniasis (VL, also known as kala-azar) is a major public health problem and, if left untreated, is fatal in most cases. Every year 50,000 to 90,000 new VL cases occur worldwide, with more than 95% of the reported cases in 2017 occurring in 10 countries, one of which is Nepal.^1^

Since 2005, under the initiative of the WHO, a VL elimination program has been underway in India, Bangladesh, and Nepal.^2^ Nepal and Bangladesh have already achieved the target of less than one case per 10,000,^3^ whereas India has 37 blocks had not reached this target in 2019.^4^

The annual number of VL cases in Nepal was 218 in 2018, which is 10 times lower than in 2006.^5^ This success was possible due to program activities involving early case detection by fever camps, index case monitoring, and proper case management combined with indoor residual spraying (IRS) for vector control. New VL cases are, however, now occurring in previously nonendemic areas with no control programs. New foci districts contributed to approximately 54% of the total VL cases in the country in 2018.^6^ The VL cases are in the Terai region, hill and mountain districts, and clustered in some villages but sporadic in many districts. The VL cases also include children who have no history of travel to VL-endemic areas.

Implementation of ongoing interventions may no longer be cost-effective for the government when the disease burden is substantially reduced and fewer cases are identified by active case detection. At the same time, the government must maintain a level of intervention to ensure sustainable success in the future so that VL does not return as a public health problem. New VL foci and areas with low VL endemicity are particularly challenging for the program. Insecticide resistance is another threat. Therefore, adequate strategies for VL case detection and vector control are needed to support the elimination program during the consolidation and maintenance phase so that new transmission foci in low VL endemic areas can be dealt with efficiently and sustainably.

A recent intervention trial found that insecticidal wall painting (IWP) with paint containing pyrethroid and insect growth inhibitors was effective for sandfly control, not inferior to IRS, and highly accepted by the community.^7^^,^^8^ The insecticidal paint can be managed by the community because of its simplicity, feasibility, and acceptance.^7^

Previous studies have also shown that early case detection is important for interrupting the spread of VL and PKDL.^9^^,^^10^ This is of particular importance in new VL foci, where local transmission is occurring.^11^

Interventions including early case detection by the index case–based approach and sandfly control through IRS or IWP could be important for combating VL in new foci and low VL endemic areas in Nepal where there is less than one VL case per 10,000 people. The feasibility and cost of these interventions needs to be determined through implementation research before they can be recommended, however. Palpa and Surkhet districts are two new VL foci in the hills, with reports of VL cases from 10 and 6 villages, respectively, in 2017. Therefore, this study was conducted to determine feasibility, effectiveness, and acceptability of sandfly control interventions and early VL case search strategies in low new VL foci in Nepal.

## MATERIALS AND METHODS

### Ethics statement.

Ethical approvals were obtained from the WHO Ethics Review Committee (Protocol ID: B60063) and the Nepal Health Research Council (Ref. 2337). Written consent was obtained from household heads before any vector control interventions or household interviews were conducted.

### Study areas and duration.

**Figure 1.**
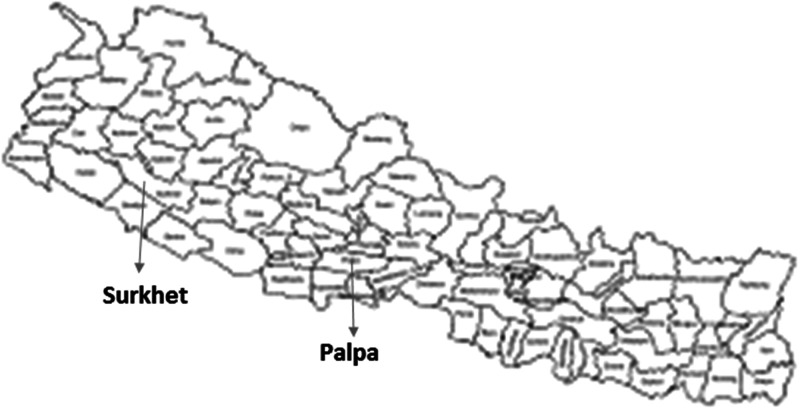
Location of study area.

In Nepal, VL cases have been reported from 64 of 77 districts. Among those districts, 18 are VL endemic program districts, whereas 46 districts are low-endemic new foci districts. In program districts, VL elimination activities including diagnosis and treatment at district hospitals, vector control, and active case detection are available, whereas in new foci districts, active case detection and vector control activities do not exist. All districts with an annual case load below 1 per 10,000 based on case reports in 2015 were identified as low-endemic new foci areas. The study was conducted in Palpa and Surkhet districts from June 2018 to June 2019. Only villages with new VL cases (index cases) reported during the study period were included in the study. The locations of the study districts are shown in Figure 1.

### Study design.

The study was an intervention study using quantitative and qualitative research methods to compare the adoption and outcomes of two intervention packages. Ten villages from Palpa and six villages from Surkhet districts where new VL index cases were reported were included in the study. The villages were randomly assigned to one of the two intervention packages so that seven localities where the index case came from received the IRS intervention package, and the other nine localities received the IWP intervention package. The screening survey for secondary cases was conducted in houses around the house of the index case within a 100-m radius. The entomological measurements and household interview survey was conducted in a subsample of these households (HHs). We performed information education and communication activities in the locality.

### Sample size and sampling.

#### Sample size for selection of district/sub-district for comparison of delay in early response with intervention.

The number of localities (i.e., neighborhoods or villages where the index case was living) was calculated based on the following assumptions: In a previous study in new foci districts of Nepal, the delay between diagnosing VL and starting response actions was 24 days.^12^ We aimed to reduce the delay to 6 days (difference of 18 days) assuming an SD of 9, ratio 1:1, alpha = 0.05 and power 90%. In addition, the required number of localities (or districts assuming one case per district) was six in each intervention group, and therefore 10 from each group would give sufficient power of detecting the difference.

#### Sample size for efficacy of the intervention (sandfly density reduction).

The sample size was calculated based on the assumptions of a 55% reduction achieved by the intervention. The average sandfly density in the control area after intervention would be 5.0/night/household with SD = 5.0 (considering over dispersion in sandfly data), and average sandfly density in the intervention area after intervention would be 2.25/night/household with SD = 2.25. With a power of 80% and 5% level of significance, using a two sample mean test, the required number of household would be 32 per intervention package for sandfly density measurements. We included 36 HHs per intervention package for sandfly density measurements. We had 10 index neighborhoods/villages for each package and HHs within 100-m radius of the index case, per locality. We selected six houses randomly in each study locality out of the 60 where we conducted the entomological measurements. The houses in the study villages were similar in structure with walls made of stone and mud. The houses in the hilly areas are usually scattered, and in cases with less than 6 households within the 100-m radius, houses outside this radius were included in the study.

#### Sample size for community peoples’ acceptance survey.

The sample size was calculated based on the assumption that 80% of people would accept the intervention package, precision ±10% and with 95% confidence interval of the estimate. The minimum number of sample size per intervention packages was 61. We interviewed 277 community people from Palpa and 171 from Surkhet.

### Index case–based activities and intervention packages for sandfly control.

Once a new VL case was identified, a screening survey was conducted for new VL/PKDL cases in the households around an index case. If a suspected case was identified, a referral form was filled and the case was transported to the nearest district hospital. District health staff were retrained on standard operating procedures about early response activities that were implemented in the locality of the index case. Training of community people on VL/PKDL transmission risk, protection, and vector control through IRS and insecticidal wall paint (IWP) was conducted. The IWP Inesfly 5AIGRNG (Inesfly Corporation, Valencia, Spain) contains alpha-cypermethrin 0.7%; D-allethin 1.0% and pyriproxyphen (0.063%). The formulation is vinyl paint with an aqueous base, with the active ingredients residing within CaCO_3_ and resin microcapsules, allowing a gradual release of active ingredients. Microcapsules range from one to several hundred micrometers in size. Vector control by indoor residual spraying with alpha-cypermethrin was conducted in households of 10 villages and IWP performed in the households of another 10 villages.

We set in place a system to collect and manage adverse events (AEs) should the painters complain of any AE during the painting. The research supervisor from the study team observed and reported any AEs of household members during the painting time as well as within the study period.

Trained field research assistants (FRAs) also visited the community to observe the intervention activities conducted by the health staff as well as by the community people. The observations were made using the structured observation checklist.

### Sandfly density measurement activities.

For sandfly surveillance, six households were selected for the sandfly density measurement. The only exception was the village from Surkhet, Gumi, which had only three households in the particular hill. The household with a VL case in the past and households around the index case were selected for sandfly density measurement. Sandfly density was measured by CDC light traps (Miniature Incandescent Light Trap, Model 1012, JW Hock Company, Gainesville, FL) on 2 consecutive nights. Light trap collections were made indoor between 18:00 and 06:00 on the next morning. Care was taken so that the lowest part of the light trap was 2 inches above the ground and 2 inches away from any wall. Sandfly densities were determined at baseline, 1 month, 9 months, and 12 months after intervention by placing the CDC light traps in the same households. Collected specimens were transported to the laboratory for examination under a binocular dissecting microscope. Sandfly species were morphologically identified by an entomologist according to the Lewis key.^13^ Total sandfly density was calculated as the number of sandflies including both female and male sandflies per household per CDC light trap per night.

### Awareness activities on VL in the communities.

People from intervention villages were invited to the nearest public place to inform them about visceral leishmaniasis, transmission risk of VL, protection from VL, VL vectors and their control, and advantages and potential risks of intervention packages. People from nearby villages also participated in the awareness session.

### Interview of household head and district program officers.

Interviews were conducted with the household head 1 month after the intervention. Trained FRAs listed all the study households for each index case village and performed the randomization to select the households to be interviewed. Only subjects who agreed to participate and freely signed the consent form were included in the study. The FRAs used the structured questionnaire to conduct the interview on perception, acceptability, and adverse events of the intervention packages.

District program officers from Palpa and Surkhet were interviewed with structured questionnaire on their awareness on VL elimination activities.

### Data management and analysis.

A well-checked data entry program was designed using SPSS Version 22. All the data were entered into this electronic system. Descriptive analysis was performed.

**Figure 2.**
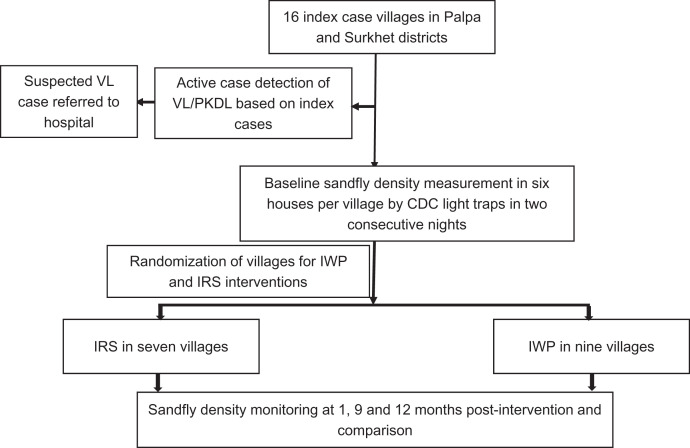
Flowchart of study procedures.

For cost analysis, costs attributable to effectiveness of the vector control interventions, reduction of sandfly density by interventions were calculated separately for comparison among the two types of intervention. Costs were broadly classified into fixed cost (that does not vary with output) and variable cost (that varies with output). The total cost of individual inputs was calculated multiplying the quantity (*q*) of individual input used by corresponding price (*p*) and summing up.^14^ Overall, total cost of each intervention was calculated separately. The average or unit cost was calculated dividing total cost of each intervention by the households that received the vector control intervention (IWP/IRS). Figure 2 provides a flowchart of study procedure.

## RESULTS

### Epidemiology of VL, household screening for febrile illness, and vector control interventions.

We identified the villages for active case detection and sandfly control interventions based on the reports of VL cases in 2017–2018. In Palpa, 10 villages reported a single case of VL and were included for intervention. Five villages each were randomly allocated to IRS and IWP. In Surkhet, six villages had reported VL cases during 2017–2018, three villages reporting one case each, two villages with two cases and one with three cases. Two of the villages were randomly selected for IRS and four villages for IWP (Table 1).

**Table 1:** Households with screening for febrile illness and vector control interventions

	Palpa		Surkhet		Total
	IRS	IWP	IRS	IWP	
No. of visceral leishmaniasis cases	5	5	5	5	20
No. of villages	5	5	2	4	16
No. of households screened during baseline and covered by intervention	186	88	50	90	414
Total population screened during baseline	1,027	483	292	400	2,202
No. of febrile cases identified during baseline and tested for rK39	0	1	0	0	1
No. of positive cases during baseline	0	1	0	0	1
No. of households screened 1 month after intervention	198	86	84	74	442
Total population screened 1 month after intervention	1,014	492	462	377	2,345
No. of febrile cases identified 1 month after intervention and tested for rK39	2	0	0	0	2
No. of positive cases 1 month after intervention	0	0	0	0	0

IRS = indoor residual spraying; IWP = insecticidal wall painting.

All of the households of the selected villages were included in the sandfly control interventions and were screened for febrile cases in which 1,221 males and 1,124 females were included. Among the screened population, we were able to detect one new case from Palpa with fever more than 2 weeks and rK39 positive who was referred to Palpa Mission Hospital and confirmed as VL positive. No skin lesion cases typical of PKDL were detected during the screening in either districts (Table 1).

A total of 186 households were treated with IRS and 88 households with IWP in Palpa. In Surkhet, 50 households were treated with IRS and 90 households with IWP. In IRS villages, two rounds of spraying was conducted after six months of first spraying. In total, 274 households in Palpa and 140 in Surkhet were screened for febrile cases for VL and skin lesion for PKDL (Table 1).

### Effectiveness of vector control interventions.

We measured sandfly density before and after IWP and IRS intervention. It was found that the total sandfly density including male sandflies was low in both interventions after one, nine and twelve months of interventions as compared with baseline. One, nine, and 12 month sandfly data has been provided in Table 2. Study in the period of 3 and 6 month could not be carried out due to winter season since the vector density remains zero in winter. We had carried out the 2^nd^ cycle of IRS hence the relative reduction of the 9th and 12th month density is realistic. In IWP intervention villages, sandfly density was 1.46 per household per CDC light trap per night at baseline before intervention while it was 0.36 after twelve months of intervention. Similarly, in IRS intervention villages, sandfly density was 0.75 per household per CDC light trap per night at baseline before intervention while it was 0.28 after twelve months of intervention. The sandfly densities at one, nine and twelve months after interventions were significantly lower as compared with sandfly density at baseline for both interventions (Table 2).

**Table 2:** Sandfly density before and after interventions

Time of measurement	Total sandfly density*		*P* value of IWP comparing with baseline	*P* value of IRS comparing with baseline
	IWP (*n* = 60)	IRS (*n* = 54)		
At baseline	1.46	0.75	–	–
After 1 month of intervention	0.14	0.17	< 0.001	< 0.001
After 9 months of intervention	0.28	0.31	< 0.001	< 0.001
After 12 months of intervention	0.36	0.28	< 0.001	< 0.001

IRS = indoor residual spraying; IWP = insecticidal wall painting.

* Per household per CDC light trap per night.

The percentage reductions in sandfly density after one, nine and twelve months of IWP intervention were 90%, 81%, and 75% respectively compared with baseline. The corresponding percentage reductions with IRS were 81%, 59% and 63% respectively. The relative reduction of sandfly densities by IWP as compared with IRS after one, nine and twelve months of interventions were 117%, 138% and 234% respectively (Table 3).

**Table 3:** Reduction of sandfly density by IWP compared with IRS

	IWP	IRS
Reduction after 1 month compared with baseline*	−1.32	−0.58
Reduction after 9 months compared with baseline*	−1.18	−0.44
Reduction after 12 months compared with baseline*	−1.10	−0.47
Percent reduction after 1 month	−90.4	−77.3
Percent reduction after 9 months	−80.8	−58.6
Percent reduction after 12 months	−75.3	−62.7
Relative reduction (%) after 1 month	116.9	–
Relative reduction (%) after 9 months	137.7	–
Relative reduction (%) after 12 months	234.0	–

IRS = indoor residual spraying; IWP = insecticidal wall painting.

* Number of sandflies per CDC light trap per night.

### Acceptability and operational issues of vector control interventions.

In Palpa and Surkhet districts, provisions for insecticide, spray pumps and trained sprayers were made available by the Government Epidemiology and Disease Control Division. For IWP, local people were trained and involved. Both IWP and IRS were accepted by the people (Palpa: IWP-100%; IRS-100% & Surkhet: IWP-100%; IRS-90%). The reported adverse events due to IWP were cough (7.4%), dizziness (7.4%), headache (7.4%), itching (7.4%), sneezing (3.7%) and due to IRS were cough (12.8%), headache (15.4%), itching (17.9%), sneezing (2.6%). In Palpa, 271 people and in Surkhet, 177 people (including from villages outside the intervention area) participated in the awareness session. District program officers were well aware of the activities related to the VL elimination program.

### Costs of vector control interventions.

We sprayed 236 households (IRS) and painted 178 households (IWP). The cost per household treated was USD 10.3 for IRS (being USD 20.6 per year for 2 rounds of spraying) and 32.8 for IWP. The overall cost per house treated was however lower with IWP when applied every 2 years instead of every 6 months for IRS (Table 4).

**Table 4:** Operational costs (in U.S. dollars) of vector control interventions in hilly districts

	IRS	IWP
Total number of HHs sprayed/painted	236	178
Travel cost	495.5	1,659.7
Daily allowance for painter/sprayer	654.5	1,163.6
Cost of materials	409.1	2,500
Logistics for spraying/painting	632.5	353.7
Total cost of intervention	1,927.6	5855
Cost per household protected	10.3	32.8
Cost of intervention per house per 2 years	41.1*	32.8

HHs = households; IRS = indoor residual spraying; IWP = insecticidal wall painting.

* Two rounds of IRS per year would imply the 4-fold costs per 2 years.

## DISCUSSION AND CONCLUSIONS

This implementation study assessed the effectiveness of sandfly control interventions IRS and IWP in new VL foci in hilly districts of Nepal. Further, combined screening of febrile illness was also conducted and people were provided education on VL and its preventive measures. This study highlights that sandfly density was reduced significantly after both IWP and IRS as compared with baseline in hill districts and could be useful for sandfly control in hilly districts for VL elimination program. There was sporadic distribution of VL cases in hilly districts and we could detect only one VL case in a screening of population around index cases of VL.

VL, once confined to the tropical Terai regions of Nepal, is moving towards the hills and the mountains of the country including the Palpa and Surkhet districts.^15^ The people of Palpa and Surkhet districts were screened for the febrile diseases for VL and skin lesions for post–kala-azar dermal leishmaniasis (PKDL). After screening a population of 2,202 at the baseline, 2,345 individuals were followed during 12 months with only one VL case detected. In a previous cross-sectional screening of 52,277 populations in VL-endemic Saptari district, no cases of VL and PKDL were detected.^7^ In a previous study, two leprosy cases were detected through screening of 15,583 households with a population of 97,032, but no VL cases were detected in the VL-endemic Sarlahi district.^16^

The VL program requires effective vector-control strategies that could work in both high-and low-endemic areas. IRS and IWP were compared as vector-control measures in this study. This intervention was conducted in hill districts with relatively low temperature (25°C) and humidity. In most villages in the district, the majority of houses and surrounding plots were made of mud and stone. The grassy ground has plants, mainly flowers, banana, and palm trees. Farm animals live within the house surroundings in cattle sheds indoors or outdoors. Previous studies in Terai districts, which has relatively high temperature and humidity, showed that IRS is effective up to 6 months. Therefore, we measured the efficacy of IWP and two rounds of IRS at 1, 9, and 12 months to maintain the same design and to observe its effects in hill districts. The results showed that although sandfly densities increased at 9 and 12 months compared with 1 month after IWP and IRS, the reduction in sandfly density was found significantly low compared with baseline. The sandfly species in hilly districts were the same as in endemic areas. Compared with baseline, the sandfly density was significantly reduced after both interventions. Reduction of the sandfly density was, however, more prominent with the IWP than IRS, as previously reported.^7^ Insecticide resistance may not be the reason for the increase of sandfly density with time because these were new VL foci, and IRS and IWP were performed and for the first time. The reason may be the decrease of residual effect of the insecticides in IRS and slower in IWP.

Sixty-six households were selected for surveying acceptability of interventions (27 from IWP and 39 from IRS). No severe adverse or unusual events were reported by the community during and after IWP or IRS. The interventions were well accepted by the community and suggested such interventions should be performed frequently to control the vector density. In a study conducted in the VL-endemic Saptari district of Nepal, IWP was more acceptable than IRS and bed-net impregnation.^7^

It was found that the Palpa district had reported three VL cases and the Surkhet district had reported one within the previous 6 months. The cases were diagnosed following the national guidelines within 3 to 5 days after arrival at the hospital. Palpa previously reported the cases to the Epidemiology and Disease Control Division (EDCD) every week (if detected), and Surkhet reported to EDCD in their monthly report. Palpa did not have a program for VL and hence had not performed any VL control activities. The district had not received any insecticide, spray materials, or workforce for the insecticidal spray. Because the district did not have any vector control or other programs, the program officer did not visit the community or perform any activity once a case was detected. The scenario was different in the case of Surkhet, which had received support from EDCD for VL control and had sufficient insecticide, spray materials, and labor for the IRS spray program.

Different costs were analyzed for the different interventions, and the cost per household was only 10.3 USD for IRS compared with 32.8 USD for IWP. Although the cost per house treated with IRS was lower, IWP lasts longer for up to 2 years compared with IRS that lasts only 6 months, making IWP more cost-effective. Efficacy of 24 months for IWP was shown by Ghosh et al. in Bangladesh (2021, personal communication). The 2-year protection cost per household including insecticide, paint, and labor cost is 41.1 USD with IRS compared with 32.8 USD with IWP. In addition to IRS or IWP, other effective alternative vector-control methods including durable wall lining (DWL) and bed net impregnation with slow-release insecticide (ITN) have been tested for sandfly control,^17^ but DWL has limited availability in the market.

The major limitations of this study include lack of control villages for testing the efficacy of sandfly control interventions. Because of the low number of households in villages in hilly areas, we were not able to keep these controls. This study did, however, include baseline measurements before intervention and was able to compare IRS with IWP directly, confirming previous studies^7^^,^^8^ that IWP, which lasts for 2 years, could be more effective over the longer period than IRS, which should be applied twice per year and therefore may not be feasible in low-endemic areas. Because of the low number of sandflies in the study area, we could not analyze the reduction in only *Phlebotomus argentipes* density and could not perform bioassay tests to determine the mortality effect of the insecticidal interventions (IWP and IRS). Further, we had not evaluated insecticide resistance of sandfly, and side effects of insecticides to the mammals.

In conclusion, local transmission of VL has recently become established in hilly districts of Nepal. Sandfly density reduction was more effective and cost-effective with IWP than IRS. Active case detection and better sandfly control with IWP or IRS including sandfly surveillance during elimination efforts can contribute to VL control in the consolidation and maintenance phase. Future operational research is needed to identify transmission dynamics of the disease in newly reported VL districts and capacity building of the rapid response team, involving the provincial and municipalities’ health staff for the sustainability of the elimination with continued sandfly surveillance by health workers and implementation of VL interventions when needed.
